# Genetic Modification for Improving Seed Vigor Is Transitioning from Model Plants to Crop Plants

**DOI:** 10.3389/fpls.2017.00008

**Published:** 2017-01-18

**Authors:** Xiaolin Wu, Fen Ning, Xiuli Hu, Wei Wang

**Affiliations:** State Key Laboratory of Wheat and Maize Crop Science, Collaborative Innovation Center of Henan Grain Crops, College of Life Sciences, Henan Agricultural UniversityZhengzhou, China

**Keywords:** *Arabidopsis*, cereal crops, genetic modification, protein l-isoaspartyl methyltransferase (PIMT), rice, reactive oxygen species (ROS), seed vigor and longevity, transgenic seeds

## Abstract

Although seed vigor is a complex physiological trait controlled by quantitative trait loci, technological advances in the laboratory are being translated into applications for enhancing seed vigor in crop plants. In this article, we summarize and discuss pioneering work in the genetic modification of seed vigor, especially through the over-expression of protein L-isoaspartyl methyltransferase (PIMT, EC 2.1.1.77) in seeds. The impressive success in improving rice seed vigor through the over-expression of *PIMT* provides a valuable reference for engineering high-vigor seeds for crop production. In recent decades, numerous genes/proteins associated with seed vigor have been identified. It is hoped that such potential candidates may be used in the development of genetically edited crops for a high and stable yield potential in crop production. This possibility is very valuable in the context of a changing climate and increasing world population.

## Introduction

Seed vigor is a complex physiological trait that is necessary to ensure the rapid and uniform emergence of plants in the field (Ventura et al., 2012), essentially including the seed longevity, the tolerance of environmental stresses by germination, and the ability to withstand prolonged storage and CDT. This trait is controlled by many QTLs that are located on different chromosomes, as found in the model plant *Arabidopsis* (Clerkx et al., 2004) and in crop plants such as rice (Cui et al., 2002; Miura et al., 2002), *Medicago truncatula* (Vandecasteele et al., 2011), and maize (Han et al., 2014) and is also affected by environmental factors during seed development, harvest, and storage.

Orthodox seeds, such as cereal seeds, undergo desiccation at the end of the maturation process on the mother plant and maintain their vigor over prolonged time periods (Rajjou et al., 2012). Because of their desiccation tolerance during dry storage, orthodox seeds are most commonly used in agriculture. For example, only three crop species (wheat, rice, and maize) account for more than 50% of all calories consumed by of the global population (Macovei et al., 2012). In addition to economic and ecological importance, high-vigor seeds are necessary for seedling establishment and sustainable crop productivity, especially under unfavorable conditions (Rajjou et al., 2012; Ventura et al., 2012). High-vigor seeds can improve seed germination and seedling emergence, increase crop yield and reduce the cost of agriculture production. With the widespread application of modern mechanized precision sowing technology for grain (e.g., maize, wheat) production, high-vigor seeds have become particularly important. In addition, for seed germplasms conserved in gene banks around the world, seed vigor and longevity may affect the regeneration cycle of accessions stored in seed banks. Seeds in long-term storage, especially under high-temperature and high-moisture conditions, will eventually lose their viability (Ventura et al., 2012). High-vigor seeds can survive a prolonged storage time.

However, the seed vigor trait is often excluded from traditional breeding programs, which are mostly directed toward high yield. To increase the vigor of commercial seed lots, the seed industry practices various invigoration treatments, especially physical priming methods (review in Ventura et al., 2012; Araújo et al., 2016). In fact, the potential of GM technology for enhancing seed vigor has been proposed as the most effective, economical and sustainable approach (Clerkx et al., 2004; Vandecasteele et al., 2011; Han et al., 2014). In the context of classical breeding, the application of GM technology to agriculturally important crops will play an increasingly important role in solving some fundamental challenges that face agriculture, natural resources and the environment. In this article, we summarize and discuss some pioneering work in the GM of seed vigor, especially through the approach of genetic engineering the PIMT (EC 2.1.1.77) in seeds. GM for improving seed vigor is just transitioning from model plants to crop plants. Promisingly, numerous genes/proteins associated with seed vigor, identified over the decades, may be used for the creation of genetically edited crops for a high and stable yield potential in crop production. This possibility is very valuable in the context of a changing climate and increasing world population.

## Physiological, Biochemical, and Genetic Bases of Seed Vigor

Seed vigor is a complex physiological trait involving regulatory networks that integrate genetic programs, metabolic signals, and hormonal signaling pathways (Rajjou et al., 2012). Many QTLs located on different chromosomes have documented associations with seed vigor. The various candidate genes identified within these QTLs are mainly involved in the glycolytic pathway, protein metabolism and signal transduction (Cui et al., 2002; Miura et al., 2002; Vandecasteele et al., 2011; Han et al., 2014). Seed vigor also has a close relationship with seed maturity degree, harvest time, and storage period: it has a maximum at physiological maturity and then decreases during storage (Sun et al., 2007). Carbohydrates, proteins, and mRNAs stored during seed development on the mother plant assist with hormone signaling pathways, especially the ABA signaling pathway, to regulate seed germination and influence seed vigor (Rajjou et al., 2012). ABA participates in regulating the expression of some seed genes in the mother plant during seed dehydration, such as LEA proteins, and inhibits the germination of developing seeds (Williams and Tsang, 1991). During storage, the seed will always deteriorate through a series of changes, such as the accumulation of ROS, lipid peroxidation, loss of cellular membrane integrity, enzyme inactivation, weak energy metabolism, and DNA degradation (Kibinza et al., 2006; Parkhey et al., 2012; Ventura et al., 2012; Xin et al., 2014; Yin et al., 2014; Kong et al., 2015; Ratajczak et al., 2015).

The loss of seed vigor is a complex normal biological phenomenon. In research on the mechanism of seed vigor change, CDT is the main way to simulate the seed aging process because aging naturally is time-consuming. Proteomics analysis displays a similar proteome characterization between artificial and natural aged *Arabidopsis* seed (Rajjou et al., 2008). However, a recent study reported substantial differences in scutellum nuclear content and morphology between the viability loss of accelerated and naturally aged wheat seed (Ahmed et al., 2016). Numerous studies have been performed on the process of seed deterioration in various plant species (e.g., Catusse et al., 2008, 2011; Galpaz and Reymond, 2010; Han et al., 2014; Nagel et al., 2014). However, the underlying mechanism remains unclear. It has become increasingly accepted that ROS damage to DNA (Vanderauwera et al., 2011), proteins (Rajjou et al., 2008) and membrane lipids (Roqueiro et al., 2010) plays a role in seed aging. ROS are continuously generated during seed development, storage and germination and exist in a state of dynamic equilibrium in cells under the action of free radical scavenger enzymes. Thus, the accumulation of ROS could be a common mechanism in seed deterioration. As a countermeasure, seed vigor has evolved a sophisticated mechanism (protection, detoxification, and repair) to protect macromolecules from ROS damage (review in Rajjou et al., 2012; Ventura et al., 2012). The possibility of restricting ROS accumulation may be a promising step toward successfully engineering seed vigor in crops.

## Potential Candidates of Genes/Proteins Associated with Seed Vigor Trait

Under natural conditions, it is very rare to acquire high-vigor seeds through natural variation. Traditional breeding has made great progress in crop improvement; however, the process is time-consuming, and the genetic resources regarding seed vigor are limited. Promisingly, with the development of global omics approaches, such as genomics, transcriptomics and proteomics, numerous potential candidates (genes/proteins) involved in seed vigor have been identified with high efficiency in recent decades (**Table 1**), though few have been detected in the identified QTLs associated with seed vigor (Cui et al., 2002; Miura et al., 2002; Vandecasteele et al., 2011; Han et al., 2014). These potential candidates may be used in breeding programs and/or in biotechnological approaches to improve seed vigor and crop yields.

**Table 1:** Candidate proteins/genes for improving seed vigor in plants.

Plant species	Target proteins/genes	Reference
Repair proteins/genes		
*Arabidopsis thaliana*	AtLIG6, AtLIG4, AtOGG1	Waterworth et al., 2010; Chen et al., 2012
*Medicago truncatula*	MSR, MtOGG1, MtFPG, MtTFIIS	Macovei et al., 2011a,b; Châtelain et al., 2013
Protective proteins/genes		
*A. thaliana*	ATEM6, PLDα1, LEA14, XERO1, RAB18, HSP70, HSP 20, HSP17.7	Gallardo et al., 2001; Manfre et al., 2006, 2009; Devaiah et al., 2007; Hundertmark et al., 2011
*Oriza sativa*	OsHSP18.2	Kaur et al., 2015
*Triticum aestivum*	HSPs	Helm et al., 1989
*Zea mays*	HSP18, HSP 17.2, HSP 16.9, LEA-3, EMB564, *PR2*, *Opaque2*, *MT1*	Revilla et al., 2009; Wu et al., 2011
*Glycine max*	*PLDα*	Lee et al., 2012
*Helianthus annuus*	*HaHSFA9*	Prieto-Dapena et al., 2006
*Nelumbo nucifera*	*NnHSP17.5*	Zhou et al., 2012a
*Beta vulgaris*	HSP17, PP2A, 14-3-3, Glycine betaine	Catusse et al., 2008, 2011
*M. truncatula*	HSP 18.2, HSP17.4, GroEL, RPN1, sHSP20	Yacoubi et al., 2011; Châtelain et al., 2012
Detoxification proteins/genes		
*A. thaliana*	SSADH, *MSD1*, *CAT1*, *HPT1*, *APX4*, AtDLAH, RBOH-B, MST, *VTE*	Bouché et al., 2003; Sattler et al., 2004; Rajjou et al., 2008; Müller et al., 2009; Xi et al., 2010; Seo et al., 2011; Wang et al., 2014
*O. sativa*	OsALDH7, ACCase, PI3K	Shin et al., 2009; Talai and Sen-Mandi, 2010; Liu et al., 2012
*Hordeum vulgare*	PER1	Stacy et al., 1999
*Z. mays*	2-Cys Prx BAS1, TPX, GST, GLO, *SOD4*, *CAT3*	Revilla et al., 2009; Wu et al., 2011
*N. nucifera*	NnANN1, *NnMT2a, NnMT2b, NnMT3*	Chu et al., 2012; Zhou et al., 2012b
*Nicotiana tabacum*	*CuZnSOD, APX*	Lee et al., 2010
*M. truncatula*	Annexin, SOD, Trx, AhpC, 1-Cys Prx, GST, Prx, MSR	Yacoubi et al., 2011, 2013; Châtelain et al., 2013
Others		
*A. thaliana*	*eIFiso4F*, *RSL1*, *Gln1*, *Gln2*	Lellis et al., 2010; Bueso et al., 2014; Guan et al., 2015
*Beta vulgaris*	ICL, SAM, Cys synthase, caleosin	Catusse et al., 2008, 2011
*G. max*	Tu1, Tu2, 1-a	Wang et al., 2012
*O. sativa*	*OsLOX*	Suzuki and Matsukura, 1997; Wang et al., 2008


### Repair Proteins

The formation of isoAsp, arising from both the deamidation of L-asparaginyl residues and the isomerization of L-aspartyl residues, is a frequent chemical modification that alters protein structure and leads to a loss of function (Lowenson and Clarke, 1992). The PIMT counteracts such damage by catalyzing the conversion of isoAsp to normal Asp in a variety of organisms, including plants (reviewed in Clarke, 2003). The PIMT-mediated protein repair mechanism represents a good example that has been successfully engineered for enhanced seed vigor (see below: case of PIMT, **Table 2**). For orthodox seeds, DNA damage, caused by ROS stress, occurs during seed dehydration and storage, leading to vigor loss. It is generally recognized that enhanced seed vigor and successful priming depend on DNA repair mechanisms activated during imbibition (Ventura et al., 2012). In *Arabidopsis*, the plant-specific DNA ligase VI (AtLIG6 and AtLIG4) is an important determinant of seed vigor and longevity under adverse germination conditions; *atlig6* and *atlig6::atlig4* mutants show significant hypersensitivity to CDT, displaying delayed germination and reduced seed vigor (Waterworth et al., 2010). A bifunctional DNA glycosylase/apurinic/apyrimidinic lyase, AtOGG1, is involved in base excision repair for eliminating 8-oxo-G from DNA, and the over-expression of *AtOGG1* enhances seed longevity and abiotic stress tolerance (Chen et al., 2012). These DNA repair pathways represent potential targets for the generation of crops with improved seed vigor traits.

**Table 2:** Physiological consequences of altering PIMT accumulation in plant seeds.

Plant species	Methodology	Main findings and altered seed traits	Reference
*A. thaliana*	T-DNA insertion line with increased *PIMT1* expression and transgenic lines with altered *PIMT1* expression	The physiological role of *AtPIMT1* in seed vigor and longevity has been established in *Arabidopsis*.	Ogé et al., 2008
		The higher PIMT1 amount in *pimt1-1* seeds correlates with lower isoAsp accumulation *in vivo* and increases both seed longevity and germination vigor, and *vice versa*.	
		Germination % after 8 days storage (40°C, 15–20% humidity): 52 and 25% for WT seeds; 80 and 50% for the *pimt1-1* mutant seeds, monitored at 4 days after sowing.	
*Cicer arietinum*	Seed-specific Over-expression of *CaPIMT1* and *CaPIMT2* in *Arabidopsis*	The role of *CaPIMT2* in seed vigor and longevity has been elucidated.	Verma et al., 2013
		*CaPIMT2* enhances seed vigor and longevity by repairing abnormal isoAsp in the seed nuclear proteome.	
		Germination % after 4 days of CDT, control seeds, 10–14%; *CaPIMT1* and *CaPIMT2* transformed seeds, 80–90%.	
*O. sativa*	Overexpressing *OsPIMT1* lines and *OsPIMT1* RNAi lines	The role of *OsPIMT1* in seed vigor and longevity has been elucidated.	Wei et al., 2015
		Germination % after 21 days of CDT, overexpressing *OsPIMT1* transgenic seeds, increased 9–15%; *OsPIMT1* RNAi lines, rapid loss of germination.	
	Transgenic rice and *Arabidopsis* lines with altered expression of *OsPIMT1* and *OsPIMT2*	The PIMT-mediated protein repair mechanism during seed development and aging in rice has been elucidated, i.e., OsPIMTs repairs antioxidative enzymes and proteins that restrict ROS accumulation, lipid peroxidation, and so on, thus contributing to seed vigor and longevity.	Petla et al., 2016
		Transgenic rice overexpressing *OsPIMT1* and *OsPIMT2* exhibits improved seed vigor and longevity.	
		Germination % after 4 days of CDT, control seeds, 8% (maximum); *OsPIMT1*, *OsPIMT2*, and *ΔOsPIMT2* transformed seeds, 43–48%.	


### Protective Proteins

Protective molecules such as LEA proteins and HSPs are generally associated with desiccation tolerance and longevity and are accumulated in the maturation phase during seed development. These stress-related proteins may also play a role in seed vigor.

Transgenic *Arabidopsis* seeds over-accumulating a HSF exhibit enhanced accumulation of HSPs and improved tolerance to aging (Prieto-Dapena et al., 2006). Knockout mutation in *ATEM6* of the *Arabidopsis* group 1 LEA family resulted in a premature phenotype, demonstrating that ATEM6 protein is associated with water retention/loss during seed maturation; however, it might not be required in mature seeds for viability or efficient germination (Manfre et al., 2006, 2009). Dehydrins are LEA proteins that accumulate during seed maturation and in response to abiotic stresses in vegetative tissues. A twofold reduction in seed-specific dehydrin (LEA14, XERO1, and RAB18) by RNAi reduced seed longevity and viability in *Arabidopsis* (Hundertmark et al., 2011). Phospholipase D, which cleaves phospholipids and generates phosphatidic acid (PA), is involved in the early stages of seed deterioration. The accumulation of PA in seeds triggers damage at the level of cellular membranes and storage lipids. Depletion of the *Arabidopsis* PLDα1 gene, encoding a member of the lipid-hydrolyzing phospholipase D family, resulted in seeds with lower levels of lipid peroxides and increased tolerance to aging (Devaiah et al., 2007).

### Detoxification Proteins

This class of proteins performs the degradation and/or elimination of endogenous and exogenous toxins, such as ROS. In particular, to eliminate ROS, cells develop a number of ROS scavengers such as superoxide dismutase, peroxidase, and vitamins. Enhanced seed longevity has been reported through the elimination of ROS by over-accumulated ROS scavengers in transgenic seeds (e.g., Lee et al., 2010).

Three genes (*NnMT2a*, *NnMT2b*, and *NnMT3*) from sacred lotus that encode metallothioneins, cysteine-rich small proteins involved in ROS scavenging, were highly expressed in germinating sacred lotus seeds and dramatically upregulated in response to high salinity and oxidative stresses (Zhou et al., 2012b). Moreover, transgenic *Arabidopsis* seeds overexpressing *NnMT2a* and *NnMT3* displayed a remarkably improved resistance to accelerated aging treatment, indicating their significant roles in seed germination vigor (Zhou et al., 2012b).

The mitochondrial SSADH is one of the three enzymes involved in the GABA shunt. In plants, the role of the GABA shunt in protection against oxidative stress has been demonstrated (Bouché et al., 2003). The presence of SSADH in dry seeds suggests that the GABA shunt is involved in the control of seed longevity or/and germination. Mutations in the OsALDH7 gene resulted in seeds that were more sensitive to artificial aging conditions and accumulated more malondialdehyde than wild-type seeds, implying that this enzyme plays a role in maintaining seed viability by detoxifying the aldehydes generated by lipid peroxidation (Shin et al., 2009).

## Genetic Modified Seeds for Enhanced Vigor: Case of PIMT

In seeds, proteins are prone to aging damage during normal aging and CDT. To date, a successful approach to enhanced seed vigor involves enhancing the accumulation of PIMT in seeds. However, no specific proteins have been assigned to the identified QTLs associated with seed vigor. The history of this effort provides an excellent example of how scientific problem solving can be brought to bear on applications in agriculture.

Mudgett and Clarke (1993, 1994) first discovered PIMT activity in plants and proposed that PIMT might be involved in seed survival by preventing isoAsp accumulation in the proteins of aging and stressed seeds. PIMT has since been detected in a wide range of plants and cloned in *Arabidopsis*, wheat, chickpea and rice, and the numbers are still increasing. In plants, PIMT is encoded by two different genes (*PIMT1* and *PIMT2*) (Xu et al., 2004), which display distinct expression patterns but similar biochemical properties (Thapar et al., 2001). Later, Ogé et al. (2008) validated the role of this enzyme in both seed vigor and longevity by altering the expression of PIMT1 in *Arabidopsis*. Their findings implicate PIMT1 as a major endogenous factor that limits isoAsp accumulation in seed proteins, thereby improving seed traits such as longevity and vigor. Recently, the role of PIMT in seed vigor and longevity has been evaluated in chickpea (*Cicer arietinum*) (Verma et al., 2013) and rice (*Oriza sativa*) (Wei et al., 2015; Petla et al., 2016). Notably, transgenic rice constitutively overexpressing *OsPIMT1* and *OsPIMT2* exhibited improved seed vigor and longevity (Petla et al., 2016).

Although the seed vigor trait depends on a wide range of physical, chemical, molecular and QTLs, the PIMT repair pathway improves seed vigor in rice by restricting the formation of deleterious isoAsp and repairing damaged proteins, not through direct DNA or lipid protection (Petla et al., 2016). This finding implies the efficacy of making high-vigor rice seeds through a target-gene approach. However, it remains to be observed whether this approach can work in the field or whether other single-gene manipulations can also produce such effects. Moreover, the effect of enhanced PIMT expression on other seed traits, e.g., nutrient value, potential health risk as food and feed, and plant phenotypes, must be extensively evaluated. In addition, the exploitation of such PIMT-mediated improvement of seed vigor in other important crops could have a huge impact on the agricultural economy. The successful case of over-expressed PIMT enhancing seed vigor proves a good guide for other potential candidates.

## Concluding Remarks and Perspective

Currently, achieving food supply security with limited arable land is a major global challenge due to the changing climate and increasing global population. The approach of modifying PIMT in seed tissues provides a rational means of creating high-vigor seeds for crop production. Its application to important cereals such as wheat, rice, and maize may have a dramatic impact on global food security. Despite substantial progress, many questions still remain. The possible effect of enhanced seed vigor obtained by the over-expression of PIMT and other proteins on the nutritional value of crops is unclear. It remains to be assessed whether a GM seed with enhanced vigor shares similar health and nutritional characteristics with its conventional counterpart.

While numerous potential candidates (genes/proteins) associated with seed vigor are available, their roles in improving seed vigor must be validated by reverse genetics on large-scale samples before translation into application in agriculturally relevant crop species. The rapid development of new genome-editing techniques enables the precise modulation of traits of interest with unprecedented control and efficiency. Among the current genome-editing tools, CRISPR is easy, rapid and inexpensive, exhibiting a broad applicability of plant genome editing for the development of designer crops (review in Khatodia et al., 2016). However, it is important to remember that the safe use of GM food or feed requires an assessment of health risks and environmental effect (Araki and Ishii, 2015).

At present, there are no reports on the application of CRISPR in manipulating seed vigor in plants. Genome-editing techniques represent a promising tool for manipulating the accumulation of proteins associated with seed vigor in a seed-specific manner and should greatly reduce the time needed to obtain valuable crop varieties. Thus, the creation of such transgenic seeds and their subsequent application in agriculture is crucial for better feeding a rapidly growing population in a changing climate.

Seed quality is the basis of agricultural production. High-quality seeds are an unremitting pursuit for every seed producer. GM technology is an effective, economical and sustainable way to improve seed vigor, change seed color or shape, or boost nutrient components and other agronomic traits for crops. The application of GM technology will sharply change the face of agriculture.

## Author Contributions

WW and XH conceived the article. FN and XW collected references and analyzed the data. XW, FN, and WW revised the manuscript. All authors contributed in manuscript writing, and approved the final manuscript.

## Conflict of Interest Statement

The authors declare that the research was conducted in the absence of any commercial or financial relationships that could be construed as a potential conflict of interest.

## Abbreviations

CDT: controlled deterioration test
GABA: γ-aminobutyric acid
GM: genetic modification
HSF: heat stress transcription factor
HSPs: heat shock proteins
isoAsp: L-isoaspartyl residues
LEA: late embryogenesis abundant
OsALDH7: rice aldehyde dehydrogenase 7
OsPIMT1: rice protein l-isoaspartyl methyltransferase
PIMT: protein l-isoaspartyl methyltransferase
PLD: phospholipase
QTLs: quantitative trait loci
ROS: reactive oxygen species
SSADH: succinic-semialdehyde dehydrogenase
